# The Impact of Obesity on Total Hip Arthroplasty Outcomes: A Retrospective Matched Cohort Study

**DOI:** 10.7759/cureus.27450

**Published:** 2022-07-29

**Authors:** Vikram A Aggarwal, Senthil Sambandam, Dane Wukich

**Affiliations:** 1 Orthopaedics, University of Texas Southwestern Medical Center, Dallas, USA

**Keywords:** costs, retrospective database, outcomes, total hip arthroplasty, complications, obesity

## Abstract

Aim

Previous research has shown that obesity is associated with worse postoperative outcomes. We aim to determine how rates of specific complications after total hip arthroplasty (THA) align with obesity status. We hypothesize that obese patients would have higher rates of complications and cost and thus have worse outcomes than non-obese patients.

Methods

Data were collected from a large commercial insurance database between 2011 and 2020. Patients underwent a hip replacement under current procedural terminology (CPT) and International Statistical Classification of Diseases (ICD-9/ICD-10) codes. Obese (defined as having a BMI of 30 kg/m^2^ or higher) and non-obese patients were matched on age, gender, Charlson Comorbidity Index (CCI), and Elixhauser Comorbidity Index (ECI). Standardized complications and costs in one year were compared using unequal variance t-tests.

Results

Under CPT codes, 61,462 obese (45% male) and 61,462 non-obese patients (45% male) underwent a hip replacement. Obese patients had significantly higher rates of surgical site infection (SSI) (OR=1.193, p=0.0001), deep vein thrombosis (DVT) (OR=1.275, p=0.001), wound complication (OR=1.736, p<0.0001), hematoma (OR=1.242, p=0.0001), pulmonary embolism (OR=1.141, p=0.0355), UTI (OR=1.065, p=0.0016), and opioid prescriptions (OR=1.17, p<0.0001), and significantly lower rates of arrhythmia (OR=0.907, p<0.0001), congestive heart failure (CHF) (OR=0.863, p<0.0001), cardiac arrest (OR=0.637 p<0.0001), pneumonia (OR=0.795, p<0.0001), and transfusion (OR=0.777, p<0.0001). Furthermore, obese patients were significantly more likely to undergo revision within 10 years (OR=1.172, p<0.0001). Under ICD codes, 31,922 obese (45% male) and 31,922 non-obese patients (45% male) were included. Obese patients did not have a significant difference in total cost or drug cost.

Conclusions

Obese patients had significantly higher rates of infection, venous thromboembolic event, wound complication, hematoma, and opioid prescriptions but significantly lower rates of cardiac issues, pneumonia, and transfusion, after hip replacement. Additionally, there was no significant difference in total or drug cost. Therefore, this study did not support our hypothesis that obese patients have worse outcomes than non-obese patients, as there neither was a clear significant increase in complication rates nor a significant increase in costs. However, further research should be done to better understand the complex relationship between obesity and postoperative outcomes.

## Introduction

Total hip arthroplasty (THA) is used to treat advanced hip arthritis and is one of the most common orthopedic surgeries in adults [1]. Despite its high success rate, various complications can worsen the patient’s quality of life and sometimes even require a revision, further encumbering the healthcare system [2]. Obesity, defined as a BMI of 30 kg/m^2^ or higher, has increased globally by 27.5% in adults and 47.1% in children over the last 30 years [3]. While obesity has various systemic effects, ranging from the kidney to cardiovascular health [4], it has specifically been shown to adversely affect bones and surrounding soft tissues biochemically and biomechanically and be a risk factor for osteoarthritis [5-6]. However, there remains controversy as to what role obesity plays in postoperative outcomes and complication rates after THA [7]. Nevertheless, as obesity becomes more prevalent, there will be a greater need for safe, effective THA with minimal complications and associated costs. Previous studies have examined the relationship between obesity and postoperative complications after THA [7,8]. Many of these studies have shown that obesity is linked to higher infection rates, dislocation, and deep vein thrombosis (DVT). However, other studies have shown that obesity has no negative effect on postoperative outcomes. Even so, these studies did not compare many complications together with a large sample size in patients with and without obesity as we have. Furthermore, to the best of our knowledge, no study has examined postoperative complications in a large, multicenter, matched cohort of patients with and without obesity undergoing THA. Therefore, we aim to examine and compare specific, prospectively selected complications and costs associated with treatment between these two groups. We hypothesize that patients with obesity would have higher complication rates and higher costs, therefore worse outcomes than patients without obesity.

## Materials and methods

Data source and study population

This retrospective study queried the PearlDiver Patient Records Database (www.pearldiverinc.com, Colorado Springs, CO, USA) for all patients who underwent a THA using current procedural terminology (CPT) and International Statistical Classification of Diseases (ICD-9/ICD-10) codes between January 2011 and January 2020. The PearlDiver database contains hospital and physician billing records along with prescription medication records for 91 million distinct patients from all US states and territories and a broad range of payer types, such as commercial insurance, including Humana and United Healthcare, Medicare, Medicaid, self-pay, and other databases like the National Inpatient Sample. Patient information from all sources is de-identified and is compliant with the Health Insurance Portability and Affordability Act (HIPAA). Therefore, the study did not require review by our institutional review board (IRB). We included patients who underwent THA during the period specified above based on CPT-27130 and ICD-9-P-8151 codes (both codes for THA) and who maintained at least one year of postoperative follow-up in the PearlDiver database. Patients who did not have medical records at least one year prior and one year after THA were excluded from the study. THA was recorded in 221,742 patients, and patients were further divided based on whether they were also listed as having the comorbidity of obesity. Group 1 (study group) was comprised of patients with obesity, and group 2 (control group) was comprised of patients without obesity.

Patient characteristics

Patients were examined based on the following characteristics: patient age, gender, Charlson Comorbidity Index (CCI), Elixhauser Comorbidity Index (ECI), and length of stay. Due to the fact that these comorbidities increase the risk for peri- and postoperative complications, we sought to compare our two groups as previously described. Bellwether-PearlDiver was used to generate propensity-matched populations from both Group 1 and Group 2 based on age, gender, CCI, and ECI. This resulted in each group having the same number of patients with similar age, gender, CCI, and ECI distributions (n=61462 for each group) based on CPT code CPT-27130. Additionally, data on length of stay and total and drug costs were provided by ICD-9 codes and not CPT codes. A similarly matched but smaller group (n=31922 for each group) based on ICD-9 code ICD-9-P-8151 was investigated. Consequently, the data in Table 1 was obtained solely using CPT codes. As demonstrated in Tables 1-2, the mean age, percentage of males, and comorbidity indices were very similar between CPT and ICD-9 codes.

Post-operative complications

The following postoperative complications were assessed between the two groups over the periods of 30 days, 90 days, and one year: dislocation, surgical site infection (SSI), myocardial infarction (MI), anemia, arrhythmia, congestive heart failure (CHF), acute kidney injury (AKI), cardiac arrest, deep venous thrombosis (DVT), wound complication, hematoma, pneumonia, pulmonary embolism (PE), blood transfusion, and UTI. These complications were chosen to be examined before data collection began. The odds ratio of complications for each group were assessed and compared, along with the 95% confidence intervals and p-value for each complication.

Total and drug costs

Both the total and drug costs were calculated over the periods of 30 days, 90 days, and one year. The total cost includes all costs from the initial arthroplasty till January 2020. The drug cost includes costs of prescription orders from the initial arthroplasty till January 2020. The means and SDs of both groups' total cost and drug cost were then calculated and compared, along with the p-value for the difference between the two groups for each span of time.

Statistical methods

Patient characteristics were described using mean (SD) for continuous variables and frequency (proportion) for categorical variables. Two-independent sample t-tests for unequal variances (continuous variables) and a test of significance were used to identify any differences between the two groups.

## Results

**Figure 1:**
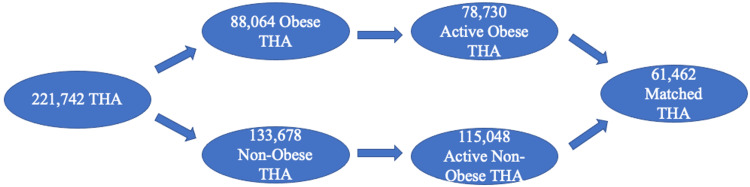
Patient selection schematic based on CPT-27447. CPT: Current procedural terminology.

As shown in Figure 1, 221,742 patients underwent THA during these nine years based on CPT-27447, and 88,064 (40%) had comorbidity of obesity. However, only 78,730 (obese) and 115,048 (non-obese) had records at least one year prior to and after THA. Therefore, these two patient populations were matched to have 61,462 patients in each group, with the remaining patients from both groups excluded from the study.

As demonstrated in Table 1, the mean age was 64 years, 45% of the patients were male, the mean CCI was 1.6, and the mean ECI was 4.5.

**Table 1:** Total hip arthroplasty patients' characteristics based on CPT codes. CPT: Current procedural terminology.

Characteristic	Obesity	Non-obesity
Age (y), mean (SD)	64.21 (+/-9.86)	64.09 (+/-9.93)
Male, N (%)	27701 (45%)	27701 (45%)
Charlson Comorbidity Index, mean (SD)	1.62 (+/-1.95)	1.61 (+/-1.94)
Elixhauser Comorbidity Index, mean (SD)	4.55 (+/-2.90)	4.51 (+/-2.89)

**Figure 2:**
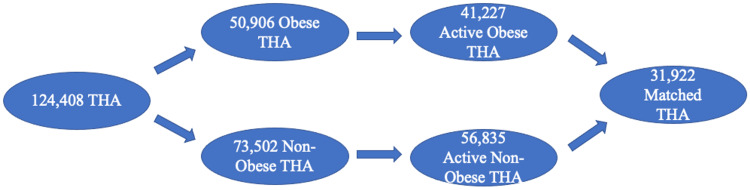
Patient selection schematic based on ICD-9-D-8154. ICD: International Statistical Classification of Diseases.

As shown in Figure 2, 124,408 patients underwent THA during these nine years based on ICD-9-P-8154, and 50,906 (41%) had comorbidity of obesity. However, only 41,227 (obese) and 56,835 (non-obese) had records at least one year prior to and after THA. Then those two patient populations were matched to have 31,922 patients in each group, with the remaining patients from both groups excluded from the study.

Between the matched groups, the mean age was 64 years, 44% of the patients were male, the mean CCI was 1.6, the mean ECI was 4.4, and the length of stay was 2.99 and 3.11 days for obese and non-obese patients, respectively (Table 2).

**Table 2:** Total hip arthroplasty patients' characteristicss based on ICD-9 codes. ICD: International Statistical Classification of Diseases.

Characteristic	Obesity	Non-obesity
Age (y), mean (SD)	64.31 (+/-9.62)	64.38 (+/-9.74)
Male, N (%)	13971 (45%)	27701 (45%)
Charlson Comorbidty Index, mean (SD)	1.57 (+/-1.89)	1.57 (+/-1.90)
Elixhauser Comorbidty Index, mean (SD)	4.37 (+/-2.64)	4.35 (+/-2.63)
Length of stay (d), mean (SD)	2.99 (+/-2.46)	3.11 (+/-2.30)

During the first 30 days after THA, obese patients experienced significantly higher rates of SSI, AKI, wound complication, hematoma, UTI, and opioid prescription than non-obese patients. Surprisingly, the complication rates of arrhythmia, CHF, cardiac arrest, pneumonia, and transfusion were significantly lower for obese than non-obese patients after 30 days (Table 3).

**Table 3:** Odds ratio of complications within the first 30 days from THA. Significant differences are highlighted in bold. THA: Total hip arthroplasty; SSI: Surgical site infection; MI: Myocardial infarction; CHF: Congestive heart failure; AKI: Acute kidney injury; DVT: Deep venous thrombosis; PE: Pulmonary embolism.

Complication	Obesity (Group 1)	Non-obesity (Group 2)	Odds ratio	Lower CI	Upper CI	P-value
Dislocation N (%)	238 (0.387%)	254 (0.413%)	0.937	0.785	1.118	0.4699
SSI N (%)	644 (1.048%)	459 (0.747%)	1.407	1.288	1.635	<0.0001
MI N (%)	558 (0.908%)	617 (1.004%)	0.903	0.805	1.014	0.0838
Anemia N (%)	719 (1.170%)	744 (1.211%)	0.966	0.871	1.071	0.5109
Arrhythmia N (%)	3811 (6.201%)	4142 (6.739%​​​​​​​)	0.915	0.874	0.957	0.0001
CHF N (%)	738 (1.201%​​​​​​​)	848 (1.380%​​​​​​​)	0.869	0.787	0.959	0.0055
AKI N (%)	1127 (1.834%​​​​​​​)	997 (1.622%​​​​​​​)	1.132	1.04	1.235	0.0045
Cardiac arrest N (%)	41 (0.067%​​​​​​​)	72 (0.117%​​​​​​​)	0.57	0.388	0.835	0.004
DVT N (%)	182 (0.300%)	197 (0.321%)	0.923	0.755	1.13	0.4404
Wound complication N (%)	406 (0.661%​​​​​​​)	196 (0.319%​​​​​​​)	2.079	1.752	2.466	<0.0001
Hematoma N (%)	470 (0.765%​​​​​​​)	395 (0.643%​​​​​​​)	1.191	1.042	1.363	0.0106
Pneumonia N (%)	532 (0.866%​​​​​​​)	741(1.206%​​​​​​​)	0.715	0.64	0.8	<0.0001
PE N (%)	229 (0.373%)	207 (0.337%)	1.107	0.917	1.336	0.2914
Transfusion N (%)	1531 (2.491%​​​​​​​)	2050 (3.335%​​​​​​​)	0.740	0.692	0.792	<0.0001
UTI N (%)	1658 (2.700%​​​​​​​)	1542 (2.509%​​​​​​​)	1.077	1.004	1.156	0.0378
Prescribed Opioids N (%)	39337 (31.761%​​​​​​​)	37492 (30.126%​​​​​​​)	1.137	1.111	1.163	<.0001

Within the first 90 days after THA, obese patients continued to have significantly higher odds of experiencing SSI, AKI, wound complication, hematoma, opioids prescription, along with PE, which had not been increased at 30 days. Arrhythmia, CHF, cardiac arrest, pneumonia, and transfusion were significantly decreased in obese patients. The dislocation decreased significantly at 90 days but not significantly at any other time frame (Table 4).

**Table 4:** Odds ratio of complications within the first 90 days from THA. Significant differences are highlighted in bold. THA: Total hip arthroplasty; SSI: Surgical site infection; MI: Myocardial infarction; CHF: Congestive heart failure; AKI: Acute kidney injury; DVT: Deep venous thrombosis; PE: Pulmonary embolism.

Complication	Obesity (Group 1)	Non-obesity (Group 2)	Odds ratio	Lower CI	Upper CI	P-value
Dislocation N (%)	401 (0.652%)	460 (0.748%)	0.871	0.761	0.996	0.0438
SSI N (%)	976 (1.588%​​​​​​​)	678 (1.103%​​​​​​​)	1.447	1.311	1.597	<0.0001
MI N (%)	859 (1.398%)	908 (1.477%)	0.945	0.861	1.039	0.2404
Anemia N (%)	923 (1.502%)	953 (1.551%)	0.968	0.884	1.061	0.4852
Arrhythmia N (%)	4919 (8.003%​​​​​​​)	5492 (8.936%​​​​​​​)	0.887	0.852	0.923	<0.0001
CHF N (%)	1028 (1.673%​​​​​​​)	1217 (1.980%​​​​​​​)	0.842	0.774	0.916	0.0001
AKI N (%)	1464 (2.382%​​​​​​​)	1327 (2.159%​​​​​​​)	1.106	1.026	1.192	0.0087
Cardiac arrest N (%)	62 (0.101%​​​​​​​)	114 (0.185%​​​​​​​)	0.543	0.399	0.741	0.0001
DVT N (%)	335 (0.545%)	289 (0.470%)	1.16	0.991	1.358	0.0651
Wound complication N (%)	722 (1.175%​​​​​​​)	375 (0.610%​​​​​​​)	1.936	1.708	2.195	<0.0001
Hematoma N (%)	616 (1.002%​​​​​​​)	497 (0.809%​​​​​​​)	1.242	1.103	1.398	0.0003
Pneumonia N (%)	862 (1.402%​​​​​​​)	1123 (1.827%​​​​​​​)	0.764	0.699	0.836	<0.0001
PE N (%)	378 (0.615%​​​​​​​)	321 (0.522%​​​​​​​)	1.179	1.015	1.368	0.0308
Transfusion N (%)	1666 (2.711%​​​​​​​)	2172 (3.534%​​​​​​​)	0.761	0.713	0.812	<0.0001
UTI N (%)	2811 (4.574%)	2672 (4.347%)	1.055	0.999	1.113	0.0548
Prescribed Opioids N (%)	42216 (68.686%​​​​​​​)	40328 (65.614%​​​​​​​)	1.15	1.122	1.177	<0.0001

One year after THA, obese patients still had a significantly higher odds of experiencing SSI, wound complication, hematoma, PE, UTI, and opioid prescription, but also a significantly higher chance of DVT. Finally, arrhythmia, CHF, cardiac arrest, pneumonia, and transfusions were all significantly lower in obese patients than in non-obese patients (Table 5).

**Table 5:** Odds ratio of complications within one year from THA. Significant differences are highlighted in bold. THA: Total hip arthroplasty; SSI: Surgical site infection; MI: Myocardial infarction; CHF: Congestive heart failure; AKI: Acute kidney injury; DVT: Deep venous thrombosis; PE: Pulmonary embolism.

Complication	Obesity (Group 1)	Non-obesity (Group 2)	Odds ratio	Lower CI	Upper CI	P-value
Dislocation N (%)	545 (0.887%)	596 (0.970%)	0.914	0.813	1.027	0.1308
SSI N (%)	1139 (1.853%)	958 (1.559%)	1.193	1.094	1.301	0.0001
MI N (%)	1810 (2.945%)	1805 (2.937%)	1.003	0.939	1.072	0.9327
Anemia N (%)	1294 (2.105%)	1323 (2.153%)	0.978	0.905	1.056	0.5666
Arrhythmia N (%)	7437 (12.100%​​​​​​​)	8102 (13.182%​​​​​​​)	0.907	0.877	0.938	<0.0001
CHF N (%)	1729 (2.813%​​​​​​​)	1995 (3.246%​​​​​​​)	0.863	0.808	0.921	<0.0001
AKI N (%)	2436 (3.963%)	2362 (3.843%)	1.033	0.975	1.094	0.2758
Cardiac arrest N (%)	150 (0.244%​​​​​​​)	235 (0.382%​​​​​​​)	0.637	0.519	0.783	<0.0001
DVT N (%)	588 (0.957%​​​​​​​)	462 (0.752%​​​​​​​)	1.275	1.129	1.441	0.001
Wound complication N (%)	916 (1.490%​​​​​​​)	531 (0.864%​​​​​​​)	1.736	1.559	1.933	<0.0001
Hematoma N (%)	787 (1.280%​​​​​​​)	635 (1.033%​​​​​​​)	1.242	1.119	1.38	0.0001
Pneumonia N (%)	1956 (3.182%​​​​​​​)	2441 (3.972%​​​​​​​)	0.795	0.748	844	<0.0001
PE N (%)	545 (0.887%​​​​​​​)	478 (0.777%​​​​​​​)	1.141	1.009	1.291	0.0355
Transfusion N (%)	1926 (3.134%​​​​​​​)	2456 (3.996%​​​​​​​)	0.777	0.731	0.826	<0.0001
UTI N (%)	5574 (9.069%​​​​​​​)	5261 (8.560%​​​​​​​)	1.065	1.024	1.108	0.0016
Prescribed Opioids N (%)	45234 (73.597%​​​​​​​)	43292 (70.437%​​​​​​​)	1.17	1.141	1.199	<0.0001

Obese patients had a significantly higher revision rate than non-obese patients across all time points (Table 6).

**Table 6:** Odds ratio of THA revision after 2, 5, and 10 years. THA: Total hip arthroplasty.

Time point	Obesity (Group 1)	Non-obesity (Group 2)	Odds ratio	Lower CI	Upper CI	P-value
2 years N (%)	1851 (3.012%)	1616 (2.629%)	1.15	1.075	1.231	0.0001
5 years N (%)	2118 (3.446%)	1834 (2.984%)	1.16	1.141	1.294	<0.0001
10 years N (%)	2194 (3.570%)	1882 (3.062%)	1.172	1.101	1.248	<0.0001

The data shows that obese patients experienced slightly lower total care costs than their non-obese counterparts. These differences were significant at 30 and 90 days but not at one year (Table 7).

**Table 7:** Total cost of care between obese and non-obese patients at 30 days, 90 days, and one year. Significant differences are highlighted in bold.

Time point	Obesity	Non-obesity	Comparison	P-value
30 days mean (SD)	$26590.88 (+/-15188.69)	$26998 (+/-15914.32)	0.985	0.0009
90 days mean (SD)	$28900.93 (+/-18737.26)	$29299.98 (+/-19619.22)	0.986	0.0086
1 year mean (SD)	$37358.23 (+/-28977.29)	$37503.45 (+/-33696.03)	0.996	0.5593

The data shows that obese patients experienced slightly lower drug care costs than their non-obese counterparts. However, these differences were not significant for any of the time points examined (Table 8).

**Table 8:** Drug cost of care between obese and non-obese patients at 30 days, 90 days, and one year. Significant differences are highlighted in bold.

Time point	Obesity	Non-obesity	Comparison	P-value
30 days mean (SD)	$303.65 (+/-877.28)	$319.53 (+/-2790.56)	0.95	0.3321
90 days mean (SD)	$689.12 (+/-1733.58)	$712.53 (+/-5163.95)	0.967	0.4426
1 year mean (SD)	$2505.95 (+/-5701.4)	$2586.83 (+/-14625.18)	0.969	0.3573

## Discussion

In a population of a large insurance database, across all examined time frames, obese patients experienced significantly higher rates of SSI and wound complications, confirming what has been observed in previous studies. Furthermore, obese patients had higher AKI, DVT, PE, and UTI rates, consistent with previous studies. These findings suggest that obesity creates significant risks for patients undergoing THA. The most common risks found in previous studies are an increased likelihood of SSI, wound complications, DVT, and PE [9-13]. Our study found that obese patients also had a significantly higher risk of developing hematomas following THA, although the existing literature has conflicting results regarding obesity and hematoma risk [14,15]. Finally, our study found that obese patients were more likely to be prescribed opioids at all time periods compared to their non-obese counterparts. This aligns with previous studies as well [16]. Interestingly, our study found significantly decreased risks of CHF, cardiac arrest, pneumonia, arrhythmia, and transfusion in obese patients compared to non-obese patients. This decrease in CHF, cardiac arrest, and arrhythmia could be explained by what is known as the “obesity paradox,” where BMI and mortality are inversely related [17,18]. This paradox theorizes that obesity may be protective and linked to higher survival rates in elderly and chronically ill populations. However, even this paradox is controversial because it could be a result of retrospective data in patient populations with already high levels of obesity-related conditions or other confounding variables. As for pneumonia, previous studies have found that obesity has been linked to a decreased mortality with pneumonia [19,20], perhaps as an extension of the “obesity paradox” described above. Finally, obese patients were found to have lower transfusion rates than non-obese patients, consistent with previous studies [21,22]. After examining complication rates, our study investigated revision rates among obese and non-obese patients. We used two, five, and ten years as time points to quantify how many patients had required a revision and found that obese patients had significantly more revisions than non-obese patients at each time point. Previous studies have also shown that obesity leads to earlier THA revision [23]. Revisions are due to various complications, most commonly septic revisions due to SSIs [23], which we have shown to be one of the most common complications among obese patients undergoing THA. Postoperative complications increase the amount of medical treatment a patient requires, resulting in a greater burden on the healthcare system and resulting in a higher associated healthcare cost. Surprisingly, obese patients had significantly lower healthcare costs at 30 and 90 days but not at one year. Perhaps this is due to higher rates of CHF, pneumonia, and transfusion in non-obese patients during the first 90 days. Previous studies have found reduced mortality from CHF and pneumonia and decreased need for transfusion [17,24,25]. This decrease in mortality could reduce the amount of healthcare intervention required and, therefore, reduce the associated healthcare costs.

The exact cause for why obesity can increase some complications after THA and not others is not yet fully elucidated. How obesity leads to osteoarthritis is also debated as to the exact role of mechanical and metabolic influence [9]. Recent research, however, has examined how adipose tissue and altered lipid metabolism characteristic of obesity lead to increased joint inflammation. Obese adipose tissue releases various pro-inflammatory cytokines, such as TNF-alpha and IL-6, both of which upregulate various matrix metalloproteinases (MMPs) that destroy cartilage, and adipokines, such as leptin and resistin, that also increase cartilage destruction. Oxidized low-density lipoproteins have been observed to cause joint inflammation and cartilage destruction, which can then be further exacerbated by free fatty acids in the serum [9]. This low-level chronic inflammation interferes with the immune system’s proper surveillance by impairing chemotaxis and altering macrophage differentiation [26], thereby explaining the greater risk of SSI in obese patients. Adverse wound healing was another complication that was significantly higher in obese patients. Adipose tissue is largely avascular and any revascularization to the damaged areas takes an indirect route, which causes substantial delay, resulting in poor oxygen and nutrient perfusion [27]. This poor perfusion causes oxidative stress, reduced collagen production by fibroblasts, and further chronic inflammation, all of which prevent proper wound healing. The surgical technique could also play a role in greater risk of SSI and adverse wound healing as the skin folds created with obesity can hinder proper surgical prep and leave some skin unsterilized. Also, greater adipose tissue can create pockets for fluid to collect and infection to grow, making effective closure more difficult. Furthermore, this chronic inflammation leads to a higher risk factor for DVT, PE, UTI, and AKI. Chronic inflammation causes activation of the prothrombotic signaling pathway in vascular cells by TNF-alpha and IL-6 cytokines and subsequent reduction in fibrinolysis [28], allowing for blood clots to become clogged in the pulmonary vasculature, renal vasculature, or other parts of the body. This same increased tendency to form blood clots could explain the decreased risk of blood transfusion in obese patients, as less blood can flow out of the vessel once it is cut. The mechanisms behind the relationship between hematomas, cardiac arrest, CHF, arrhythmias, and pneumonia in obese patients, however, are not yet fully understood. Opioid prescription was significantly higher in obese patients than in non-obese patients, and some authors hypothesize that obesity is associated with higher postoperative pain [29]. The cause of this pain is not fully elucidated as it could be due to the chronic inflammation or biomechanical burden placed on the body from obesity. With this higher opioid prescription, obese patients would be expected to have higher costs of care [30]. However, our data showed that obese patients experienced lower total and drug costs than non-obese patients at all three timepoints examined, with total costs at 30 and 90 days being significantly different. Obesity’s association with such a variety of postoperative complications, however, has been correlated with high total costs and drug costs [31]. Nonetheless, this correlation is not easily explained as there is a variety of confounding factors that are difficult to match, such as differences in economic status, access to healthcare, or other social determinants of health. As with our study, we could only match for age, gender, CCI, and ECI. However, these are not fully expansive and could interfere with calculating obesity’s effect on costs. Furthermore, the “obesity paradox” mentioned above could play a role in artificially lowering the costs of care associated with obesity during the early postoperative period. Also, healthy patients could have been placed under the category of obesity due to more muscle tissue than adipose tissue. Interestingly, obese patients had higher revision rates, yet were found to have significantly lower costs than non-obese patients after 30 and 90 days. This is not surprising because revision surgery is often performed years after the index procedure, and our study did not capture those costs. According to one study, the average time between primary and revision surgery was three years for patients aged 64 and younger and one year for patients aged 65 and older [32]. Therefore, the substantial cost of a revision would not be included in 30- and 90-day time frames, artificially lowering the total costs of care.

We recognize several limitations of this study. As this was a retrospective study with data gathered from various institutions and centers of practice, there were likely differences in surgical technique, equipment, and postoperative protocols. While this does increase the number of patients included in the study, the procedures and perioperative protocol were not standardized among all patients. Additionally, we could not determine if any procedures were done prior to the THA. Another limitation is that there are varying levels of obesity based on increasing BMIs, which the PearlDiver database did not differentiate. Those levels have been shown to affect postoperative outcomes [22,33] and could bias the results of this study. Furthermore, in order to reduce confounding variables, we matched patients on both CCI and ECI. However, this could lead to potential bias in which healthier obese patients are included in the study. This could explain some of the paradoxical results in the obese patient group. Retrospective studies rely on the accuracy of data that was previously entered. As a result, any errors or differences in data entry due to non-standardized and subjective criteria could have affected our results. The PearlDiver database records data based on CPT and ICD-9 codes, potentially limiting the data retrieval. Despite these limitations, we aimed to minimize the number of confounding variables by matching patients based on age, gender, CCI, and ECI and limit non-responder bias by only including patients who had records a year prior to and after their index THA. The large number of patients from the PearlDiver database increases the predictive power of the study, and the matching reduces the effect of confounding variables. Another strength is that all the examined complications were chosen before data collection began. This helps to increase the credibility of our results. Furthermore, none of the authors of this study neither matched the patients nor performed the surgeries, thereby further reducing the amount of selection bias. As our population ages and the prevalence of obesity increases, revision THA will likely increase. A better understanding of the postoperative complications associated with obesity encourages educating patients and families regarding the potential risks of surgery. Finally, as we transition from volume-based reimbursement to value-based reimbursement, surgeons can use the results of this study to guide decision-making in THA.

## Conclusions

Our large, matched patient population study found significantly higher rates of SSI, wound complications, hematoma, opioid prescription, revision surgery, DVT, PE, AKI, and UTI in the obese cohort. At the same time, there were significantly lower rates of CHF, cardiac arrest, arrhythmias, pneumonia, and transfusion during the first 90 days and decreased total costs during the first 90 days for obese patients. As there was neither a significant increase across complication rates nor a significant increase across costs, this study did not support our hypothesis that obese patients have worse outcomes than non-obese patients. Nevertheless, obese patients perform differently than non-obese patients following THA, and further research must be done to understand the mechanisms behind these complications better and establish the relationship between various other complications, diagnoses, or costs after THA in obese patients.
